# Targeting *KRAS* Mutant in Non-Small Cell Lung Cancer: Novel Insights Into Therapeutic Strategies

**DOI:** 10.3389/fonc.2022.796832

**Published:** 2022-02-16

**Authors:** Anne-Laure Désage, Camille Léonce, Aurélie Swalduz, Sandra Ortiz-Cuaran

**Affiliations:** ^1^ Univ Lyon, Claude Bernard Lyon 1 University, INSERM 1052, CNRS 5286, Centre Léon Bérard, Cancer Research Center of Lyon, Lyon, France; ^2^ Department of Pulmonology and Thoracic Oncology, North Hospital, University Hospital of Saint-Etienne, Saint-Etienne, France; ^3^ Department of Medical Oncology, Centre Léon Bérard, Lyon, France

**Keywords:** *KRAS G12C*, non-small cell lung cancer (NSCLC), adagrasib, sotorasib, acquired resistance

## Abstract

Although *KRAS-*activating mutations represent the most common oncogenic driver in non-small cell lung cancer (NSCLC), various attempts to inhibit *KRAS* failed in the past decade. *KRAS* mutations are associated with a poor prognosis and a poor response to standard therapeutic regimen. The recent development of new therapeutic agents (*i.e.*, adagrasib, sotorasib) that target specifically *KRAS G12C* in its GDP-bound state has evidenced an unprecedented success in the treatment of this subgroup of patients. Despite providing pre-clinical and clinical efficacy, several mechanisms of acquired resistance to *KRAS G12C* inhibitors have been reported. In this setting, combined therapeutic strategies including inhibition of either SHP2, SOS1 or downstream effectors of *KRAS G12C* seem particularly interesting to overcome acquired resistance. In this review, we will discuss the novel therapeutic strategies targeting *KRAS G12C* and promising approaches of combined therapy to overcome acquired resistance to *KRAS G12C* inhibitors.

## Introduction

*RAS* (*Rat sarcoma viral oncogene homolog*) encodes a membrane-bound protein, initially described in 1960s by Harvey (1) and Kirsten (2) as a retroviral oncogene involved in cell proliferation, differentiation and survival. *RAS* transforming properties were firstly reported in 1982 in human bladder cancer cell lines (3) and is considered the most frequently mutated oncogene in humans (*i.e.*, 19% of cancer patients harboring a *RAS* mutation) (4). Notably, the *KRAS* (*Kirsten rat sarcoma viral oncogene*) isoform represents 75% of *RAS* mutant cancers (4).

In particular, the highest frequency of *KRAS* alterations is identified in pancreatic adenocarcinoma (88%), colon and rectal adenocarcinoma (50%), and in lung adenocarcinoma (32%) (4, 5). In pancreatic carcinoma, *KRAS* mutations are predominantly found in codon G12, followed by a smaller proportion of mutations in codons Q61 and G13. A similar distribution is observed in non-small cell lung cancer (NSCLC) patients. However, in the latter, the location of mutations beyond the Q61 codon within the *KRAS* gene is more heterogeneous than in pancreatic adenocarcinoma cases (6).

In non-small cell lung cancer (NSCLC), the clinical significance of *KRAS* mutated oncogene was firstly demonstrated in 1984 (7). Since this discovery, and based on the successful development of targeted therapies in other oncogenic-driven NSCLC, many attempts to target *KRAS* in NSCLC were made in past decades. Despite thorough pre-clinical and clinical research, these attempts failed, thus considering *KRAS* as an “undruggable” alteration. The recent discovery of sotorasib and adagrasib, which specifically target *KRAS G12C*, provides new therapeutic strategies to improve patient outcomes.

In this literature review, we focus on *KRAS* mutations in NSCLC and discuss the pre-clinical and clinical development of sotorasib and adagrasib. Herein, we outline the main mechanisms of acquired resistance described to *KRAS G12C* inhibitors to present and summarize therapeutic strategies to overcome resistance.

## KRAS Biology and Mutations in Non-Small Cell Lung Cancer

### RAS Structure and Downstream Effectors

**Figure 1:**
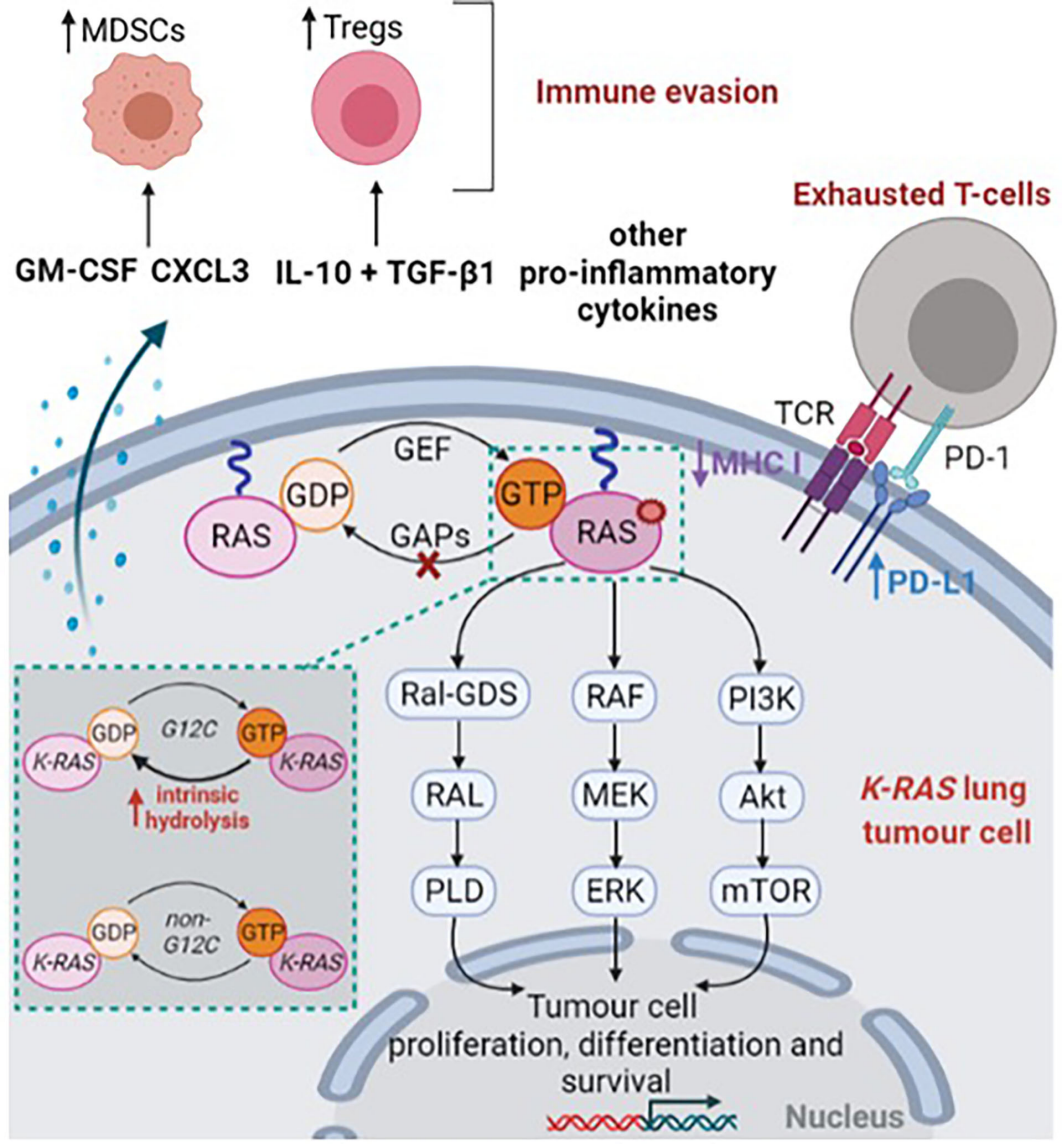
*KRAS*-mutant lung tumor cell. This figure was created with Biorender.com.

*RAS* encodes a membrane bound protein with a guanosine triphosphatase (GTPase), that is expressed in all mammalian cells (8, 9). RAS protein acts as a molecular switch, cycling between an active guanosine triphosphate (GTP)-bound state and an inactive guanosine diphosphate (GDP)-bound state (10, 11) (**Figure 1**). The cycling of RAS protein to its active form is promoted by guanine nucleotide exchange factors (GEF) while GTPase activating proteins (GAPs) contribute to maintain RAS in its inactive state through GTP hydrolysis (10, 11) (**Figure 1**). RAS cycling regulation needs the recruitment of either GEF or GAPs to the inner face of the cell membrane (8). GEF activation, which is necessary to activate RAS, is mostly related to signaling from either tyrosine kinase receptors or G-protein coupled receptors (10). Notably, EGFR (epidermal growth factor receptor) activation is known to induce wild-type RAS activation through the recruitment and the interaction of Grb2 complex (Growth factor receptor-bound protein 2) with SOS protein (Son of sevenless) (12, 13). In a lesser extent, upregulated gene expression or missense mutation might result in aberrant GEF activation (10), thereby promoting RAS activation.

RAS protein is composed of three major domains: (1) the G-domain – a highly conserved domain between RAS isoforms – which contains switch-I (aa 30-38) and switch-II (aa 59-76) loops is responsible for GTP-GDP exchange; (2) the C-terminal domain known as the hypervariable region and associated with significant variations among RAS isoforms and (3) the C-terminal CaaX box responsible for post translational modifications (10, 14–16). Of note, the C-terminal CaaX box is involved in the farnesylation of the cysteine residue; a first-step that ensures RAS localisation in the inner face of the cell membrane (16).

As a result, the binding of GTP to RAS induces a conformational change in switch-I and switch-II loops which, in turn, activates RAS (14). Consequently, activated RAS promotes RAF recruitment (17–20) and PI3K activation (21, 22) contributing to cell proliferation, differentiation and survival (**Figure 1**). Moreover, Ral GDS pathway (Ral guanine nucleotide dissociation stimulator) was also found as a key RAS effector, involved in vesicle trafficking and cytoskeletal organisation (23, 24) (**Figure 1**). In the case of somatic activating *RAS* mutant, RAS presents a reduced ability to hydrolyse GTP or to interact with GAPs, thereby leading to a permanent and constitutive activation of RAS and downstream effectors which promotes tumorigenesis (8, 9, 25) (**Figure 1**).

### KRAS Mutations in Non-Small Cell Lung Cancer and Impact on Tumorigenesis

*KRAS* is considered as a key oncogenic factor as it represents 75% of all *RAS* mutations (4). Indeed, preclinical studies on genetically engineered mouse models assessed that *KRAS* mutations predisposed to early onset lung cancer (26). The *KRAS* gene consists of 6 exons on chromosome 12 (8, 27). These point mutations mainly occur at exon 2 and exon 3 (8, 27). Indeed, 96% of *KRAS* mutations in NSCLC occur at either G12, G13 or Q61 codons (28).

*KRAS* mutations in NSCLC are often associated with tobacco history (i.e., 6.9% in never smokers vs. 32.3% and 36.9% in former and current smokers respectively) (29). As well, *KRAS* alterations are more frequent in adenocarcinoma (32%) than squamous cell carcinoma (4%) (4, 29, 30). Notably, the *G12C* mutation (i.e., mutation from amino acid glycine to cysteine) is the most frequent, as it constitutes 40% of all *KRAS* mutations in NSCLC (29). Nassar et al. recently outlined that *KRAS G12C* mutations are found in 13.8% of patients with a NSCLC (31). The *G12V* and *G12D* mutations represent 21% and 17% of *KRAS* mutations in NSCLC, respectively (29). Moreover, *KRAS G12C* mutations seem to be more frequent in women (i.e., 43.4%; *p*=0.007) and younger patients (i.e., median age 63.1 years old; *p*=0.0092) compared with other *KRAS* mutations (29, 30) and KRAS wild-type (32). Likewise, it was reported that *KRAS G12C* mutations were more frequent in black and white patients than Asian patients (*p*<0.001) (31), and are more frequent in current or former smokers (i.e., 41%), while *G12D* mutations mostly occur in non-smokers (i.e., 56%) (29). As well, *KRAS G12C*-positive patients present a higher rate of metastasis at diagnosis compared with KRAS wild-type (i.e., 94.4% vs. 88.4%) (32). Despite some reports highlighting higher frequency of brain metastasis in *KRAS* mutated NSCLC patients (33, 34), *KRAS* mutations are not considered to specifically drive brain metastasis as is the case for *ALK* rearrangements.

Notably, co-occurring alterations in non-oncogenic drivers of NSCLC are reported in about one-half of patients harboring *KRAS* mutations (35). Co-occurring mutations in *TP53* (about 40%), *STK11* (i.e., serine/threonine kinase 11; 19.8% to 28%) and *KEAP1* (i.e., kelch like ECH associated protein 1; 13% to 24%) are the most frequently reported (35–37).

*KRAS* mutations do not usually coexist in the context of *EGFR-*, *ALK-*, or *ROS*- driven NSCLC. Schmid et al. (38), reported nine cases of metastatic NSCLC which harbor ALK/KRAS co-alterations, a fraction of which (86%) were primary refractory to crizotinib (ALK inhibitor). Although *EGFR* and *KRAS* mutations typically occur in a mutually exclusive fashion in lung cancer (39, 40); anecdotal reports show evidence of co-occurrence of KRAS and EGFR mutations in lung cancer patients (41–43).

*KRAS G12C* and other *KRAS* mutations more frequently co-occurred with *MET* amplification in both localised (30) and metastatic (35, 36), treatment-naïve NSCLC. *KRAS G12C*-positive patients were also found to significantly harbor increased frequency of *ERBB2* amplification (*p*=0.002) or *ERBB4* mutations (*p*=0.025) compared with non- *KRAS G12C* patients (35).

Besides promoting tumorigenesis through downstream effectors, mutant *KRAS* cells have been found to interact with the tumor microenvironment (**Figure 1**). Firstly, *KRAS* mutations downregulate the expression of major histocompatibility class I molecules in colorectal carcinoma, thereby decreasing priming and cross-presentation to T-cells (44, 45). Similarly, *KRAS* mutations are associated with higher PD-L1 (programmed cell death ligand 1) expression in NSCLC, thus contributing to exhausted T-cells (46, 47). Notably, the proportion of PD-L1 TPS (*i.e.*, tumor proportion score) ≥50% was reported in a range of 34 (32) to 41% (48) for *KRAS G12C* patients compared with 20.4% (32) to 25.9% (48) among KRAS wild-type patients. *In vitro*, downstream effectors of *KRAS-*mutated NSCLC cell lines, including MAPK and STAT3 signaling pathways were found to promote ectopic PD-L1 expression (49, 50).

As another assessment of promoting immunosuppressive microenvironment, analyses in an *in vitro* model of *KRAS G12V* induction highlighted that *KRAS-*mutant cells enhance the secretion of TGF-β1 and IL-10, thus inducing regulatory T cells (Tregs) (51). Such results were also further confirmed in mouse models with lung tumors (51). Several reports also outlined that pro-inflammatory cytokines such as IL-6, IL-17, IL-8 and IL-22, are highly secreted by *KRAS-*mutant cells (52–55). Likewise, *KRAS* mutant cells were found to markedly secrete granulocyte macrophage colony-stimulating factor (GM-CSF) which contributes to the recruitment and activation of myeloid-derived suppressor cells (MDSCs) (53, 56). The recruitment of MDSCs was also found to be promoted by repression of interferon regulatory factor 2 in *KRAS* colorectal carcinoma, which in turn increased the expression of CXCL3, a chemokine which binds to CXCR2 receptor expressed on MDSCs (57).

### Prognostic Value of KRAS Mutations in Non-Small Cell Lung Cancer

Despite some conflicting results (32, 58, 59), *KRAS* mutations in NSCLC seem to be associated with worse prognosis (60–63). Notably, in a large meta-analysis of 43 observational studies including stage IIIB-IV NSCLC patients treated with either chemotherapy combined with or without bevacizumab and EGFR TKIs, Goulding et al found that *KRAS* mutations are associated with a significant shorter overall survival (HR=1.71; 95%CI [1.07-2.84]) and progression-free survival (HR=1.18; 95%CI [1.02-1.36]) (61). The objective response rate (RR) was also significantly lower for patients harboring *KRAS* mutations (RR=0.38; 95%CI [0.16-0.63]) (61). *KRAS* mutations were also identified as an independent worse prognostic factor in stage I disease (64). On the contrary, in a large cohort of 1039 advanced or metastatic NSCLC patients treated with chemotherapy alone or in combination with immune checkpoint inhibitor, no significant difference was observed in terms of PFS and OS between *KRAS* wild-type, *KRAS G12C* and non-*KRAS G12C* patients (32).

The *KRAS* mutation sub-type as well as co-occurring mutations seem important to take into account as they might impact prognosis and response to treatment. Indeed, a better disease-free survival for patients with stage I lung adenocarcinoma harboring *G12V*/*G12C* mutations (*p*=0.0271) was reported, compared with other *KRAS* mutations (64). Conversely, Finn et al. recently reported among a cohort of 1012 patients with a stage I-III lung adenocarcinoma that *KRAS G12C* mutations are associated with a significant shorter OS and relapse-free survival, compared with other *KRAS* mutations and wild-type *KRAS* (30). On the contrary, no significant difference was reported in terms of OS and PFS between *KRAS G12C* and non- *KRAS G12C* patients treated with chemotherapy alone or in combination with checkpoint inhibitor as first-line treatment for advanced or metastatic NSCLC (32). Notably, patients with both *KRAS* and either *STK11* (35, 65) or *KEAP1* (37) co-mutations were reported to have shorter survival.

## Sotorasib and Adagrasib: New Promising Therapeutic Strategies to Target *KRAS G12C* in NSCLC

Despite thorough pre-clinical and clinical research, *KRAS* was considered as an elusive target for a long time. Indeed, the picomolar affinity of RAS for GTP and the smooth surface of *KRAS* with a lack of well-defined hydrophobic pocket prevented either the development of GTP inhibitors or the development of *KRAS* inhibitors (66). Likewise, inhibitors of *KRAS* membrane positioning such as farnesyl transferase inhibitors (67, 68) and inhibitors of RAS membrane binding (69) failed to demonstrate clinical efficacy due to adaptative *KRAS* prenylation by geranylgeranyl transferase (70). In the same way, strategies to inhibit RAS downstream effectors or synthetic lethality approaches failed to show clinical efficacy (68, 71–74) due to either feedback activation of RAS upstream effectors or tumor heterogeneity regarding KRAS synergistic oncogenic dependencies (75).

### Discovery of KRAS G12C Inhibitors

In 2013, in the seminal work of Ostrem and colleagues, X-ray crystallographic studies revealed a new allosteric pocket beneath the effector switch-II region (76). This specific allosteric pocket was found to be located between the central β-sheet of RAS, and the α-2 (switch-II) and α-3 helices. Notably, switch-II binding pocket was only visible in RAS GDP-bound state (76). In line with these observations, a tethering approach (i.e., a fragment-based drug discovery approach) was used to screen compounds against *KRAS G12C* in the GDP-state (76).

The identification of early-stage compounds evidenced that direct targeting of *KRAS G12C* relies on covalent binding to *cysteine 12* and switch-II binding pocket region when *KRAS G12C* is in its inactive GDP-state (76). Consistently with these findings, pre-loading of GTP to *KRAS* impairs binding of these early-stage compounds to *KRAS G12C* (76). Of note, these early-stage compounds were shown to decrease cell viability and to induce cell apoptosis in *KRAS G12C* lung cancer cell lines (76). Overall, these experiments provided new strategies to target *KRAS G12C*, while sparing wild-type KRAS or other *KRAS* mutants, based on covalent binding to mutant *cysteine 12* residue and switch-II binding pocket.

Based on this promising proof-of-concept, subsequent *KRAS G12C* inhibitors with higher specificity were developed through structure-based optimization, such as ARS-853 (77, 78) and ARS-1620 (79). Janes et al. demonstrated that ARS-1620 significantly inhibits tumor growth in MIA-PaCa2 pancreatic cancer xenograft models (79).

Subsequent pre-clinical studies allowed to precise the mechanism of action of *KRAS G12C* inhibitors (77–79). Notably, these studies showed that *KRAS G12C* inhibitors trap KRAS in its inactive GDP-bound state by reducing its susceptibility to nucleotide exchange factors (77, 78), and that efficacy of *KRAS G12C* inhibitors requires intact GTPase activity (77, 78). Indeed, in engineered H358 cells (*KRAS G12C*) that express the *A59G* mutation – which abrogates RAS GTPase activity – ARS-853 failed to decrease *KRAS* GTP levels and ERK phosphorylation (77). As well, *RAS* mutations that increase nucleotide exchange activity (i.e., *Y40A*, *N116H* and *A146V*) reduced susceptibility to *KRAS G12C* inhibitors *in vitro* (77). On the contrary, *KRAS G12C* inhibition was enhanced by SOS1 inhibition (77). Consistently with these observations, Lito and Patricelli highlighted that *KRAS G12C* inhibition is dependent on the activity of upstream tyrosine kinase receptors (77, 78). Indeed, when cells are treated with EGF (Epidermal Growth Factor), *in vitro* activity of ARS-853 decreases (77, 78) while EGFR inhibition with either erlotinib, afatinib (78) or gefitinib (77) enhanced the potency of *KRAS G12C* inhibitors. Finally, it has been reported that *KRAS G12C* has a high intrinsic hydrolysis rate, compared to other *KRAS* mutations (80) (**Figure 1**). This intrinsic property might explain that *KRAS G12C* undergoes sufficient hydrolysis to enable inhibition by GDP-state selective drugs (72).

More recently, AMG-510 (81) and MRTX849 (82) were reported to have increased *KRAS G12C* inhibition activity over previous inhibitors, through enhanced interaction with the H95 residue in the α-3 helix of *KRAS G12C*. These improvements in structure-based design and biopharmaceutical optimization led to the initiation of the first-in-human trial of AMG-510 in 2018 (ClinicalTrials.gov NCT03600883).

### Sotorasib (AMG-510): Pre-Clinical and Clinical Development

In pre-clinical testing, sotorasib was found to impair cell viability in pancreatic and lung adenocarcinoma cell lines, in monolayer and spheroid models, with a high potency and high selectivity, as it had no impact on cell viability of non-*KRAS G12C* cell lines (**Table 1**) (83). Sotorasib inhibits ERK phosphorylation in multiple *KRAS G12C*-mutant *in vitro* and *in vivo* models (i.e., xenograft and syngeneic mouse models and patient-derived xenografts) (**Table 1**) (83). Besides its impact on tumor cell signaling, sotorasib was also found to restore an efficient immune tumor response (84, 86) as evidenced by increased T-cell infiltration and immune effectors including macrophages, CD103^+^ dendritic cells and CD4^+^/CD8^+^ T-cells (**Table 1**) (83).

**Table 1:** Sotorasib: synthesis of pre-clinical and clinical development.

**Pre-clinical study** (83)					
Impact on cell viability – *In vitro* (monolayer cell lines and spheroid models).		Impact on downstream effectors – *In vitro* (monolayer cell lines)	Effect of sotorasib *in vivo*		Impact on tumour microenvironment – *In vivo*
- Impaired cell viability: IC_50_≈0.006µM in NCI-H358 cell line; IC_50_≈0.009µM in MIA PaCa-2 cell line. Impaired cell viability confirmed on 22 other cell lines with heterozygous or homozygous *KRAS G12C* mutations, except for SW1573 cell line.		- Complete inhibition of ERK phosphorylation (IC_50_≈0.03µM) after 2 hours of treatment in NCI-H358 and MIA PaCa-2 cell lines.	- Maximal inhibition of ERK phosphorylation observed between 2 to 4 hours after treatment; sustained inhibition for 48 hours (NCI-H358, MIA PaCa-2 T2 and CT-26 *KRAS G12C* tumours). No inhibition of AKT phosphorylation.		- Flow-cytometry and immunohistochemistry analysis of CT-26 *KRAS G12C* tumours after 4 days of sotorasib: increased infiltration of CD3+, CD4+ and CD8+ T-cells; increased infiltration of macrophages and CD103+ dendritic cells.
		- Results confirmed in 22 other cell lines with heterozygous or homozygous *KRAS G12C* mutations.			
- Impaired cell viability on spheroid models (MIA PaCa-2, NCI-H1373, NCI-H358, NCI-H2122).		- No effect on ERK phosphorylation for non *KRAS G12C* cell lines.	- Inhibition of tumour growth in MIA PaCa-2 T2 and NCI-H358 xenograft models.		- Transcriptional analysis of CT-*26 KRAS G12C* tumours after 2 days of sotorasib: significant increased of interferon signaling, CXCL10 and CXCL11 chemokines, MHC and TLR score, NK cell score, T-cell function and cytotoxic function score
- No impact on cell viability for non *KRAS G12C* cell lines.			- Inhibition of CT26 *KRAS G12C* tumours in syngeneic mouse models; durable cure obtained in 8 out of 10 mice. No durable cure observed in CT26 *KRAS G12C* tumours of immunodeficient mice (*i.e.*, BALB/c nude mice).		
			- Inhibition of tumour growth in a colorectal cancer patient-derived xenograft model and *KRAS G12C* SCLC and NSCLC patient-derived xenograft models.		
**Clinical studies** (84, 85)					
Study	Study characteristics	Number of patients included	Clinical efficacy	Safety	Other
CodeBreaK 100 Trial, 2020 (85)	- Phase 1 - Sotorasib in patients with advanced tumours harbouring *KRAS G12C* mutations.	129 patients (*i.e.*, 59 patients with a NSCLC, 42 patients with a colorectal cancer, 28 patients with other cancers).	Sub-group analysis among patients with a NSCLC (according to RECIST 1.1*) at the time of data cut-off:	*Among the whole cohort:* - No dose limiting toxic effect	- Dose of 960 mg orally administered once daily identified for the expansion cohort
				- No treatment-related death reported	
	- Patients previously treated with systemic therapy.		- 32.2% of patients had complete or partial response (95% CI [20.62-45.64])	- Grade 3-4 toxicities according to CTCAE^†^ criteria: 11.6%	- Median time to maximum plasma concentration: 2.0 hours (range 0.3 to 6.0 hours)
	- Patients with untreated active brain metastases excluded.		- 88.1% of patients had objective response or stable disease (95% CI [77.07-95.09]).		- Mean elimination half-life: 5.5 ±1.8 hours
			- median PFS: 6.3 months	
CodeBreak 100 Trial, 2021 (84)	- Phase 2	126 patients enrolled; 124 patients evaluated for analysis	*At the time of data cut-off, March 2021:*	- Treatment related adverse-event according to CTCAE criteria: any grade (69.8%); grade 3 (19.8%); grade 4 (0.8%)	*Sub-group analysis Biomarkers:*
	- Evaluation of sotorasib (*i.e.*, administered orally at 960 mg once daily) in patients with advanced or metastatic *KRAS G12C* mutated NSCLC, previously treated with systemic therapy (*i.e.*, ≤3 previous lines of systemic therapy).	- Objective response (*i.e.*, complete or partial response): 37.1% of patients (95% CI [28.6-46.2])	- Most frequent treatment related adverse events (any grade): diarrhea (31.7%), nausea (19.0%), alanine or aspartate aminotransferase increase (15.1% respectively)		- 86 patients with PD-L1 expression assessment
				➔ PD-L1<1%: 48% response; PD-L1 1-49%: 39% response; PD-L1≥50%: 22%	- *KEAP1* mutated (*i.e.*, 20 patients): 20% response *vs.* 44% for wild-type KEAP 1
					- STK11 mutated (*i.e.*, 35 patients): 40% response *vs.* 39% for STK11 wild-type

* RECIST 1.1.: Response evaluation criteria in solid tumors; ^†^CTCAE: Common terminology criteria for adverse events.

Sotorasib, formerly AMG-510, was the first direct *KRAS G12C* inhibitor to enter clinical development (85), in a phase I/II first-in-human clinical trial (NSCLC cases in phase I=59 patients enrolled; NSCLC cases in phase II=126 patients enrolled; **Table 1**) (84, 85). In the phase II trial, sotorasib induced an objective response rate of 37.1% and a disease control rate of 80.6% (84). Among the 124 patients eligible for evaluation, 4 patients presented a complete response (84). Recently, Ramalingam et al. reported the clinical efficacy of sotorasib among patients with stable brain metastasis (n=40) included in the phase I/II CODEBREAK 100 clinical trial. In this sub-group analysis, sotorasib demonstrated clinical efficacy with a median PFS of 5.3 months and a median OS of 8.3 months (87). Sotorasib has a tolerable safety profile: 19.8% of patients experienced grade 3 while 0.8% experienced grade 4 treatment-related adverse events (84). The most frequent treatment related adverse events reported were diarrhea (31.7%), nausea (19%), increase in either alanine aminotransferase (15.1%) or aspartate aminotransferase (15.1%) levels and asthenia (11.1%) (84). In particular, as suggested in a recent report (88), sotorasib might trigger immune-related hepatitis in patients previously treated with immune checkpoint inhibitors. Sub-group analysis of the phase II cohort suggested that sotorasib either maintains or improves quality of life, physical functioning and the severity of key lung cancer-related symptoms, including cough, chest pain and dyspnea (89).

Despite small sub-group sample size, sotorasib demonstrated efficacy across a range of co-occurring mutations including *STK11* and *TP53*, whereas a lower percentage of response was observed among patients harboring a *KEAP1* co-mutation (84). Finally, although current limited data available, no predictive biomarkers of response to sotorasib have been yet identified (84, 86). Exploratory analysis of the CODEBREAK 100 trial showed that *KRAS G12C* patients who harbor a *KEAP1* mutation are likely early-progressors (i.e., patients with an event of progressive disease and PFS<3 months) whereas patients with alterations in effectors of cell cycle, WNT pathway and MAPK pathway are more prevalent in the late-progressor group (i.e., patients with an event of progressive disease and PFS≥3 months) (87). Zhao et al. reported among 43 patients treated with sotorasib - including 36 NSCLC patients - that exceptional responders (i.e., defined as a complete response or a partial response lasting more than 12 months) tend to have a lower plasma *KRAS G12C* allele frequency and a lower tumor burden at baseline compared to other patients (90). Based on the clinical efficacy and safety profile, sotorasib was recently approved by FDA for the treatment of advanced *KRAS G12C* NSCLC patients following at least one prior systemic therapy.

### Adagrasib (MRTX849): Pre-Clinical and Clinical Development

Adagrasib, formerly MRTX849, is the second irreversible and selective *KRAS G12C* inhibitor to have entered clinical trials.

Adagrasib was optimized to exhibit a long half-life (≈24 hours) and extensive tissue distribution (91, 92). Similar to sotorasib, adagrasib impairs cell viability of pancreatic and lung adenocarcinoma monolayer and spheroid models harboring *KRAS G12C* (**Table 2**) (93). Consistently with pre-clinical findings on sotorasib, adagrasib inhibits ERK phosphorylation while it has no impact on AKT activation (**Table 2**) (93). Pre-clinical studies revealed that *KEAP1* loss might constitute a potential intrinsic resistance to adagrasib as sgRNA targeting *KEAP1* were enriched following adagrasib treatment in xenograft models (**Table 2**) (93). In line with the observations reported for sotorasib, Briere et al. recently outlined, in *KRAS G12C* syngeneic and genetically engineered mouse models, adagrasib decreases intra-tumor MDSCs while it increases M1-macrophages, dendritic cells and CD4^+^/CD8^+^ T-cells (94). Interestingly, the *in vivo* efficacy of adagrasib was markedly decreased in T-cell deficient *nu/nu* mice (94).

**Table 2:** Adagrasib: Synthesis of pre-clinical and clinical development.

**Pre-clinical study** (93)								
Impact on cell viability – *In vitro* (monolayer cell lines and spheroid models).		Impact on downstream effectors – *In vitro* (monolayer cell lines)		Effect of adagrasib *in vivo*			Impact on downstream effectors – *In vivo*	
– Impaired cell viability in monolayer cell lines (*i.e.*, MIA PaCa-2, H358, H1373, H2122, SW1573 and H2030 cell lines) with IC_50_ values ranging from 10 to 975 nM. – Impaired cell viability in spheroid models (MIA PaCa-2, H1373, H358, H2122, H2030 and SW1573) with IC_50_ values ranging from 0.2 to 1042 nM. – No impact on cell viability for non *KRAS G12C* models in monolayer cell lines and spheroids.		– Inhibition of ERK phosphorylation with a maximal inhibition observed at 24 hours in MIA PaCa-2 cell line (IC_50 =_ 4.7 nM) and at 48 hours in H358 cell line (IC_50 =_ 9.2 nM). – No inhibition of AKT phosphorylation.		– Maximal inhibition of ERK phosphorylation at 6 hours after treatment (MIA PaCa-2, H1373 and H2122 xenografts). – Inhibition of tumor growth in MIA PaCa-2 and H358 xenografts. – Inhibition of tumor growth in human *KRAS G12C*-mutant cell line and patient-derived xenograft models. Tumor regression exceeding 30% volume reduction from baseline in 65% of these models after 3 weeks of treatment. No effect in non *KRAS G12C* models. – In xenograft models: *TP53*, *STK11*, *KRAS* mutant allele frequency and *CDKN2A* not identified as predictive biomarkers.			– In xenograft models, RNA seq analysis following adagrasib showed that MAPK pathway negative regulators were the most strongly decreased genes. – In xenografts, magnitude of reduction of MYC and cyclin B1 protein levels correlated with adagrasib anti-tumor activity. – In H2122 xenografts, CRISPR/Cas9 screen revealed that sgRNA targeting cell cycle, SHP2, MYC and mTOR were the top depleted sgRNA on adagrasib treatment. sgRNA targeting *KEAP1* were enriched in H2122 xenograft models treated with adagrasib.	
**Clinical study** (91, 92)								
Study	Study characteristics		Number of patients included		Clinical efficacy	Safety		Other
KRYSTAL-1 Trial, 2020 (91, 92)	– Phase I/II - Patients with advanced or metastatic solid tumors harboring *KRAS G12C* mutation. – Patients previously treated with systemic therapy.		*As of 30 August 2020:* 79 pretreated NSCLC patients.		*As of 30 August 2020*; *among 51 patients evaluable for clinical activity:* objective response (45%); disease control (96%).	– Most commonly reported (>20%) treatment related-adverse events: nausea (54%), diarrhea (48%), vomiting (34%), asthenia (28%) and increased in alanine aminotransferase level (23%). – Grade 3/4 hyponatremia according to CTCAE^†^ was reported in 3% of patients.		– Dose of 600 mg orally administered twice daily identified for the expansion cohort – Sub-population of patients with *STK11* co-occurring mutation (n=14): objective response rate of 64%. Immune transcripts (e.g., CD4 and CD8) increased after adagrasib treatment compared to baseline. – mechanistic biomarkers analyses on 3 pre- and post-treatment tumor biopsies: downregulation of K-RAS/MAPK genes including DUSP6 and SPRY4.

^†^CTCAE, common terminology criteria for adverse events.

Adagrasib is currently being tested in the KRYSTAL-1 multi-cohort phase I/II study that enrolled patients with advanced or metastatic solid tumors harboring a *KRAS G12C* mutation (NCT03785249) (91, 92). A recent preliminary report on the clinical efficacy of adagrasib confirmed an objective response rate and a disease control in respectively 45% and 96% of evaluable patients in the NSCLC cohort (NSCLC patients: n=79; **Table 2**) (91). Similar to sotorasib, nausea, diarrhea, vomiting, asthenia and increase in alanine aminotransferase level were the most frequent treatment related-adverse events reported (**Table 2**) (91). Despite small sub-group sample size, adagrasib treatment efficacy is reported regardless of the presence of concurrent mutations in *STK11* (92).

## Biological Mechanisms of Acquired Resistance to Sotorasib and Adagrasib

The objective response rates obtained for either sotorasib or adagrasib is markedly lower compared to those obtained with osimertinib or alectinib in *EGFR* and *ALK*-driven NSCLC respectively (95, 96), suggesting the presence of intrinsic mechanisms of resistance to *KRAS G12C* inhibitors.

Previous efforts to target the RAS–RAF–MEK pathway have been challenged by early adaptive feedback reactivation of signaling pathways, leading to therapeutic resistance. In KRAS-mutant cancers, trametinib (MEK inhibitor) provokes a compensatory response involving the fibroblast growth factor receptor 1 (FGFR1), resulting in signaling rebound and adaptive drug resistance (97, 98). In *KRAS G12C*-mutant models, treatment with sotorasib leads to rapid RAS pathway reactivation, followed by a significant rebound of ERK phosphorylation (average of 75% vs baseline levels) (99). In this setting, RAS-MAPK feedback reactivation was driven by intrinsic activation of various receptor tyrosine kinases (RTKs) including EGFR, HER2, FGFR and c-MET, that resulted in stimulation of wild-type RAS (NRAS and HRAS), which is not inhibited by G12C-specific inhibitors but can be counteracted by the combination of KRAS G12C and SHP2 inhibitors (99).

Interestingly, shortly after treatment, some cancer cells are sequestered in a quiescent state with low KRAS activity. The new KRAS G12C is maintained in its active, drug-insensitive state by epidermal growth factor receptor (EGFR) and aurora kinase (AURKA) signaling. In this setting, the synthesis of new KRAS G12C and its trading between the active or inactive states, modulates the divergent early response to KRAS G12C inhibition and allow cells to resume proliferation.

Initial response to *KRAS G12C* inhibition is also associated with induction of EGFR phosphorylation, following sotorasib treatment in NCI-H358 cells (83), or a marked recovery of ERK phosphorylation in adagrasib-partially sensitive H358 and H2122 cells (93). Furthermore, *IGF2* (i.e., insulin-like growth factor 2) mRNA levels were significantly higher in BEAS2B*^KRAS G12C^* cells treated with sotorasib, compared to control (100). Similarly, AURKA (Aurora Kinase A) – a cell-cycle regulator – was found to promote early adaptative resistance to ARS-1620 *in vitro* and in H358-xenograft models (101).

**Figure 2:**
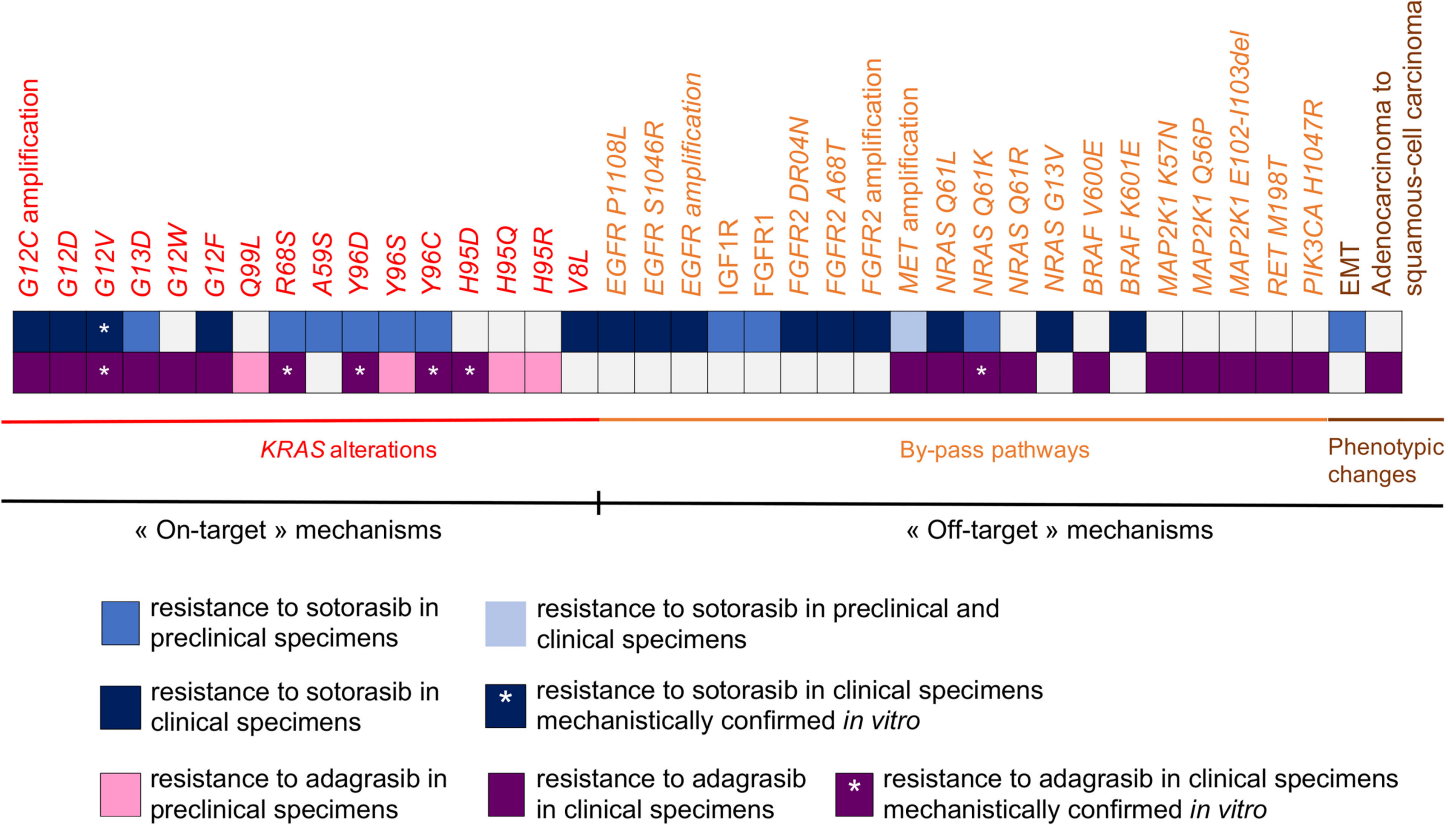
Synthesis of biological mechanisms of acquired resistance to sotorasib and adagrasib described in pre-clinical specimens and clinical specimens.

In this section, we outline the mechanisms of acquired resistance, to either sotorasib or adagrasib, described *in vitro*, *in vivo* or in the clinical setting. The mechanisms of resistance to *KRAS G12C* targeted therapies can be classified into: (1) on-target mechanisms; (2) by-pass alterations resulting in abnormal activation of downstream or connecting signaling pathways; and (3) phenotypic transformation (102) (**Figure 2**).

### On-Target Mechanisms of Acquired Resistance

Secondary mutations in *KRAS* that confer acquired resistance to *KRAS G12C* inhibitors are currently described *in vitro* and in clinical setting (103–105). Similar to the well-known *T790M* gatekeeper mutation in *EGFR*-oncogenic driven NSCLC (106), these mutations occur in switch-II binding pocket and thereby alter drug binding (107). Similar to other oncogenic-driven NSCLC (106), acquired *KRAS-*activating mutations and *KRAS* amplification mediate acquired resistance through RAS signaling pathway activation (107).

Koga et al. modelled the acquisition of mutations associated with sotorasib or adagrasib resistance, using Ba/F3 cells treated with ENU (*i.e.*, N-ethyl-N-nitrosourea) (103). Secondary *KRAS* mutations were identified in 87.3% of the 142 resistant clones generated. In sotorasib resistant clones, *KRAS G13D* was the most frequent secondary mutation (*i.e.*, 23% of *KRAS* secondary mutations) observed, followed by *R68M* and *A59S* (21,2% for each, respectively). In adagrasib*-*resistant clones, *KRAS Q99L* was the most frequent secondary mutation (*i.e.*, 52.8% of *KRAS* secondary mutations), followed by *Y96D* and *R68S* (*i.e.*, 15,3% and 13.9% respectively) (103). Of note, although *Y96D* and *A59S* were the only mutations shared between sotorasib- and adagrasib-resistant clones, only *Y96D* mechanistically proved to confer strong cross-resistance to both *KRAS G12C* inhibitors (103). Cross-resistance of *Y96D*, but also of *Y96S*, were also assessed in H358 cells (NSCLC, *KRAS G12C*) (103). Upon adagrasib treatment, the *KRAS Y96D* mutation was associated with sustained ERK and AKT activation in several *in vitro* models including MIA Pa-Ca2 (pancreatic cancer), *KRAS G12C/Y96D* NSCLC cells and the MGH1138-1 patient-derived model (104). Moreover, a higher level of active GTP-bound KRAS was observed in *KRAS G12C/Y96D* compared to *KRAS G12C* despite treatment with adagrasib (104). Secondary mutations that alter drug binding are reported in patients who progressed on adagrasib and were exogenously expressed as double-mutant alleles in Ba/F3 cell lines, to model their mechanistic impact on *KRAS G12C*-inhibitor sensitivity (105). Notably, switch-II binding pocket mutations, including *R68S*, *H95D/Q/R* and *Y96C*, conferred resistance to adagrasib in Ba/F3 cells compared to the control *KRAS G12C* allele (105). Consistently with Koga and colleagues, the *Y96C* mutation caused cross-resistance to both adagrasib and sotorasib *in vitro* (105).

These mutations were anticipated to structurally alter drug binding (103–105). Indeed, the crystal structure of KRAS G12C binding with sotorasib or adagrasib showed that the residues G13, A59, Q61, R68, Y96 and Q99 face the drug binding pocket (103). In line with these observations, analysis of *R68S*, *H95D/Q/R* and *Y96C* mutant residues were found to disrupt non-covalent binding interactions between *KRAS G12C* and adagrasib (105). Notably, *Y96D* was found to abolish the normal direct hydrogen bond with the pyrimidine ring of adagrasib, while it disrupted the water-mediated hydrogen bond between Y96 and the carboxyl-group on sotorasib (104). *Y96D* also introduced a negatively charged amino-acid which contributes to change the binding pocket towards a substantially more hydrophilic pocket; thereby reducing drug binding (104). Besides disrupting drug binding, secondary mutations such as *A59* or *Q61* might alter GTPase activity which is normally required for efficient *KRAS G12C* inhibition (103). Similarly, mutations occurring at codons 13, 59, 61, 117 and 146, that impede GTP hydrolysis or increase GDP-to-GTP nucleotide exchange (80) were also found to promote resistance *in vitro* to MRTX1257 (*i.e.*, a compound highly related to adagrasib) and sotorasib (103, 105).

These *in vitro* experiments highlight that several acquired mutations have different sensitivities to *KRAS G12C* inhibitors. Indeed, *G13D* and *A59S* secondary mutations remained partially sensitive to adagrasib while they conferred strong resistance to sotorasib (103). Likewise, *Q99L* (103) and *H95D/Q/R* (105) secondary mutations which conferred resistance to adagrasib, remained sensitive to sotorasib. Based on a positive-selection screen for mutations that confer resistance to either sotorasib or MRTX1257, in Ba/F3 cells, Awad et al. identified that mutations occurring at codons 12, 68, 95 and 96 conferred strong resistance to MRTX1257; while mutations occurring at codons 8, 9, 12, 96 and 117 were associated with a strong resistance to sotorasib (105).

Overall, these observations suggest drug-specific binding mechanisms of resistance and indicate for rationales of sequential therapeutic strategies between sotorasib or adagrasib to counteract acquired resistance.

Interestingly, recent reports in the clinical setting support the *in vitro* findings highlighting that secondary *KRAS* mutations within drug-binding pocket and activating mutations in *KRAS* mediate acquired resistance to sotorasib and adagrasib. Tanaka et al. recently reported the case of a 67 year-old woman treated with adagrasib, in the KRYSTAL-1 dose expansion cohort, who developed heterogeneous mechanisms of acquired resistance including *KRAS G12C/Y96D, G13D* and *G12V* (104).

In a recent report, genomic and histologic analyses of pre-treatment samples compared with those obtained at progressive disease on adagrasib were conducted among 38 patients enrolled in phase I KRYSTAL-1 trial (*i.e.*, 27 patients with NSCLC, 10 with colorectal cancer and 1 with appendiceal cancer) (105). This analysis revealed that, among patients with putative mechanisms of resistance to adagrasib, 53% had at least one acquired *KRAS* mutation or *KRAS* amplification (105). Consistently with *in vitro* observations, acquired mutations within switch-II binding pocket – *Y96C*, *R68S*, *H95D* – were reported among NSCLC patients. *KRAS G12D* and *G12V* activating mutations were reported in 2 NSCLC patients (105). Additional mutation in the G12 codon as well as *cis*-mutations were also described as potential mechanisms of acquired resistance to adagrasib (104, 105). Indeed, Awad et al. reported an additional mutation in *G12*, leading to a mutation from cysteine to tryptophane (i.e., G12W mutation) (105). Then, based on the observation of concurrent *G12C* and *G12V* in *cis* on the same strand (i.e., *G12F*), Tanaka et al. hypothesized that *cis* mutations resulting in *G12C* loss and conversion into another *KRAS* mutation might constitute an additional mechanism of acquired resistance (104). Finally, high-level of *KRAS G12C* amplification was reported at relapse on adagrasib in one NSCLC patient (105).

As for adagrasib, genomic and histologic analysis of pre-treatment samples compared with those obtained at progressive disease on sotorasib were recently reported among 32 NSCLC patients enrolled in CODEBREAK 100 and CODEBREAK 101 clinical trials (90). Among these, putative mechanisms of acquired resistance were identified in 78% cases. In particular, secondary *KRAS* mutations were identified in 4 patients (i.e., *G12D*, *G12V*, *G12F*, *V8L*) while 3 patients presented *KRAS* amplification (90). The impact of *G12V* secondary mutation was mechanistically confirmed in *KRAS G12V* dox-induced H358 cells as it decreased the antiproliferative effect of either sotorasib or adagrasib (90). Finally, loss of *KRAS G12C* dependency alone or concomitant to secondary *KRAS* mutations was also found to confer resistance to either adagrasib (105) or sotorasib (90).

### By-Pass Mechanisms: Activation of RTKs and RAS Downstream Signaling Pathways

Activation of by-pass signaling pathways constitutes another main mechanism of acquired resistance described in oncogenic-driven NSCLC (106), to ensure sustained signaling activation despite therapeutic pressure.

Based on a phospho-RTK array of *KRAS G12C* NSCLC H23-sensitive and H23-resistant clones to sotorasib, Suzuki et al. showed that MET and HGF (i.e., hepatocyte growth factor) were significantly upregulated in sotorasib-resistant cells (108). Notably, H23 sotorasib-resistant cells had an abnormal MET/CEP7 ratio (i.e., 2.7), indicating that *MET* amplification promoted acquired resistance in these cells. No acquired *KRAS* mutations were identified (108). Functional experiments show that MET knockdown restored sensitivity to sotorasib *in vitro* and reverted sustained ERK phosphorylation (108). RTK activation has been reported with acquisition of epithelial-to-mesenchymal (EMT) features in sotorasib-resistant cells (109). Indeed, in resistant H358 cells that display EMT features, IGF1R (i.e., Insuline-like Growth Factor Receptor 1) activates AKT pathway while FGFR1 (i.e., Fibroblast Growth Factor Receptor 1) promotes ERK rebound activation (109). In line with these observations, IGF1R and FGFR1 were found to mediate acquired resistance in H358 and LU65-sotorasib resistant cells harboring EMT features (109).

In their report, Tanaka et al. showed that several mechanisms of acquired resistance to adagrasib might co-occur in the clinical setting, thereby highlighting the intra-tumoral heterogeneity of resistant tumors. Indeed, cfDNA sequencing and droplet digital PCR (ddPCR) in plasma samples collected at disease progression on adagrasib revealed up to 10 distinct mutations affecting RAS-MAPK pathway effectors (104). In particular, these mutations include activating mutations in *NRAS* (i.e., *Q61L*, *Q61K* and *Q61R*), *BRAF V600E* and *MAP2K1* (i.e., *K57N*, *Q56P* and *E102-I103del*) (104). The impact of *NRAS Q61K* secondary mutation on adagrasib sensitivity was mechanistically confirmed in H358-engineered cells and was also found to confer resistance to sotorasib *in vitro* (90). Moreover, alterations in *RET M198T* and *PIK3CA* as well as *MET* amplification were also detected in 5 NSCLC patients with progressive disease on adagrasib (105). Similar to adagrasib, mutations affecting RAS-MAPK pathway effectors were also identified at acquired resistance to sotorasib (90). In clinical setting, these mutations included *NRAS G13V*, non *V600E- BRAF* (i.e., *K601E*), *EGFR* (i.e., *S1064R* and *P1108L*) and *FGFR2* (i.e., *A68T* and *D304N*) activating mutations (90). *MET* and *EGFR* amplifications were also detected at progressive disease on sotorasib and co-occurred in one patient. Likewise, *FGFR2* amplification was detected in one patient (90).

### Lineage Plasticity and Acquisition of Features of Epithelial-To-Mesenchymal Transition

Histologic transformation to either small-cell lung cancer or squamous-cell carcinoma is a well-known biological mechanism of acquired resistance to targeted therapies in NSCLC (110, 111). While there is no current evidence of transdifferentiation to small-cell lung cancer in the context of acquired resistance to *KRAS G12C*-targeted therapies, Awad et al. reported two cases of phenotypic changes to squamous-cell carcinoma at disease progression on adagrasib (105). Of note, initial *KRAS G12C* mutation was detected at resistance while no other *KRAS* acquired mutations occurred in these patients (105). Interestingly, *STK11* deletion seems to drive lineage transformation to squamous-cell carcinoma in *KRAS* mutated lung adenocarcinoma (112).

Epithelial-to-mesenchymal transition represents another non-genomic transcriptional reprogramming that mediates acquired resistance to targeted therapies (111). In the setting of *KRAS G12C*-mutant NSCLC, the acquisition of EMT features *in vitro* and in xenograft models was reported to mediate acquired resistance to sotorasib (109). H358 and LU65 cells generated resistant to sotorasib presented features of EMT including E-cadherin downregulation and vimentin upregulation (109). Of note, gene set enrichment analysis of H358 sotorasib-resistant cells showed an enrichment of the EMT transcriptomic signature, compared to their sensitive parental counterparts (109). Likewise, H358 sotorasib-resistant xenograft models presented an induction of vimentin. In both resistant models to sotorasib, no acquired *KRAS* mutations were detected (109).

Overall, despite the recent development of sotorasib and adagrasib, biological mechanisms of acquired resistance have yet been described in both pre-clinical and clinical specimens (**Figure 2**).

These findings highlight the diversity of *KRAS* mutations that emerge in response to *KRAS G12C* inhibitors, thereby limiting the potential development of efficient next-generation *KRAS G12C* inhibitors (103–105). Some of these mutations display differential sensitivity to either sotorasib or adagrasib, thus providing a therapeutic rationale at progressive disease (103, 105). In the clinical setting, putative mechanisms of acquired resistance to adagrasib were described in 45% cases, suggesting that other mechanisms might be implicated in resistance to *KRAS G12C* inhibitors (105). Finally, pre-clinical and clinical observations provide rationale for combined therapy to prevent or delay acquired resistance, since 41% patients were found to have more than one concurrent mechanism of acquired resistance to adagrasib (105). As for adagrasib, concurrent treatment associated alterations at progressive disease on sotorasib were found in 60% cases of patients that present putative mechanisms of acquired resistance (90).

## Targeting Acquired Resistance to *KRAS G12C* Inhibitors

As highlighted in the seminal work of Canon et al., sotorasib might improve therapeutic efficacy of targeted agents including inhibitors of upstream and downstream effectors of *KRAS G12C* (83). Analysis of synergy scores of sotorasib associated with different targeted therapies provided proof-of concept to enhance therapeutic efficacy to revert or overcome acquired resistance (83). In H358 cells, the highest synergy scores were observed for HER kinases inhibitor (i.e., afatinib), Src Homology Phosphatase 2 inhibitor (i.e., RMC-4550) and MEK inhibitor (i.e., trametinib); the latter achieving the highest synergy score in an NCI-H1373 spheroid model (83). Consistently, a significant reduction in tumor volume was observed in H358 xenograft models treated with sotorasib plus MEK inhibitor, compared to sotorasib or MEK inhibitor monotherapy (83). Interestingly, sotorasib also enhanced therapeutic efficacy of carboplatin *in vivo* (83).

In line with these observations, adagrasib was found to have enhanced efficacy when associated with other targeted agents (93). Combined therapy of adagrasib with either afatinib or RMC-4550 (SHP2 inhibitor) induced a significant greater anti-tumor efficacy compared to single-agent monotherapy, in xenograft models of NSCLC and oesophageal squamous cell carcinoma (93). Similarly, targeting downstream *KRAS* effectors such as mTOR and members of the cyclin D family might constitute an efficient approach to enhanced adagrasib efficacy *in vivo* (93).

In this section, we will focus on reported therapeutic strategies to overcome acquired resistance to either sotorasib or adagrasib.

### Structurally and Functionally Different KRAS G12C Inhibitors: The Case of RM-018

**Figure 3:**
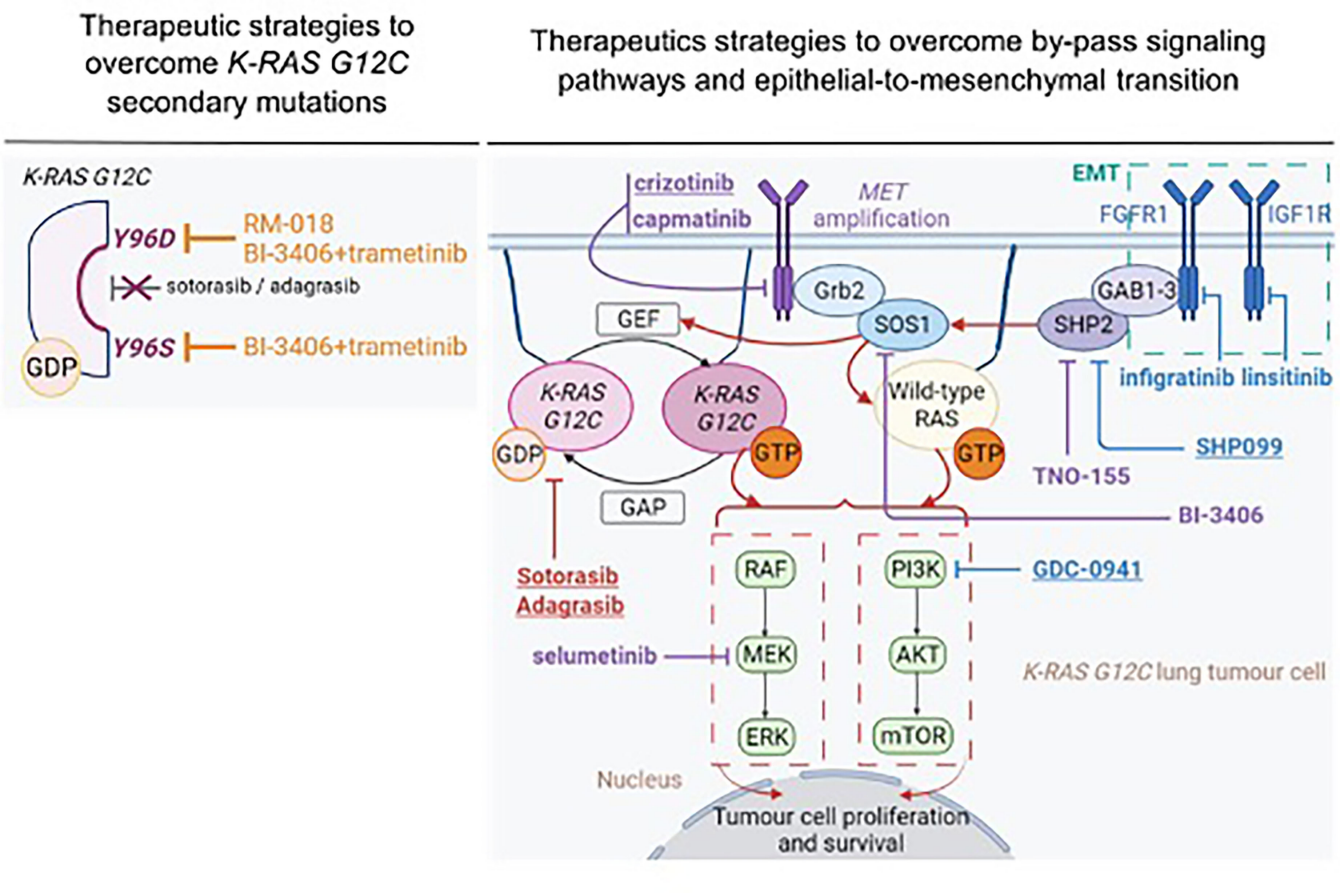
Therapeutic strategies to overcome acquired resistance to sotorasib and adagrasib. Treatment that demonstrated therapeutic efficacy in both *in vitro* and *in vivo* setting are underlined. Therapeutic strategies that demonstrated efficacy in pre-clinical specimens in the context of acquired resistance related to *MET* amplification are represented in purple. Therapeutic strategies that demonstrated efficacy in pre-clinical specimens in the context of acquisition of epithelial-to-mesenchymal features are represented in blue. This figure was created with Biorender.com.

As already outlined, *KRAS G12C/Y96D* mutations were found to confer cross resistance to sotorasib and adagrasib, through altered drug binding (103, 104). In this setting, Tanaka and colleagues sought to determine whether structurally and functionally different *KRAS G12C* inhibitors could target the *KRAS Y96D* mutation (104). RM-018 is a novel *KRAS G12C* inhibitor that exploits cyclophilin A to bind and inhibit *KRAS G12C* in its GTP-bound state (113, 114). *In vitro*, RM-018 was found to impair cell viability and to markedly decrease RAS-MAPK signaling in cell lines harboring *KRAS G12C* (113, 114). *In vivo*, administration of RM-018 in H358 *KRAS G12C*-NSCLC xenograft models resulted in dose-dependent tumor regression and was well tolerated (113, 114). Moreover, in the context of acquired resistance, RM-018 achieved *in vitro* efficacy (104). Indeed, no IC_50_ shift was observed upon RM-018 therapeutic pressure in several models including MIA PaCa-2, H358, Ba/F3 cells and the MGH1138-1 patient-derived model, harboring *KRAS G12C/Y96D* compared to *KRAS G12C* parental cells (104). In line with these observations, RM-018 also inhibited ERK activation with a high potency *in vitro* (104). Taken together, these pre-clinical data provide proof-of-concept that RM-018 could overcome *KRAS Y96D*-resistance mutation (**Figure 3**).

### Targeting RTKs Involved in By-Pass Signaling Pathways and Epithelial-to-Mesenchymal Transition

Observations *in vitro* and *in vivo* at acquired resistance to *KRAS G12C* inhibitors highlighted that cancer cells develop adaptative strategies to overcome the selective pressure and survive under treatment. This might result from *KRAS* secondary mutations or from activation of upstream effectors such as RTKs, in the context of by-pass signaling pathway or epithelial-to-mesenchymal transition. In this setting, several strategies are currently described to overcome acquired resistance based on combined therapy targeting either the specific RTKs involved in the resistance process or upstream and downstream effectors of *KRAS G12C* (115).

Some pre-clinical studies sought to determine the impact of targeting RTK-mediated acquired resistance to *KRAS G12C* inhibitors (108, 109) (**Figure 3**). Combined treatment with sotorasib and crizotinib in H23 sotorasib-resistant cells that acquired *MET* amplification, was more efficient in inhibiting ERK, AKT and MET activation and inducing apoptosis (i.e., increased cleaved-PARP and BIM expression), compared to single monotherapy (108). Moreover, combination therapy was efficient enough to eliminate RAS-GTP in similar levels as those observed in H23 parental cells. In line with these observations, the IC_50_ value of combined treatment (IC_50 =_ 0.22 µM) was markedly inferior to those obtained with either sotorasib (IC_50 =_ 69.33µM) or crizotinib (IC_50 =_ 2.68µM) monotherapy, in *MET*-amplified, sotorasib-resistant cells (108). Notably, combined treatment with sotorasib and crizotinib during four weeks, significantly decreased tumor growth in *MET*-amplified H23-sotorasib resistant xenograft models compared to monotherapy (108). Interestingly, combined treatment with sotorasib and capmatinib – a MET inhibitor that is more specific of the MET kinase – decreased cell viability with higher potency (i.e., IC_50 =_ 0.07µM) (108) (**Figure 3**).

MEK inhibitor might constitute an alternative strategy in *MET*-amplified tumors as pre-clinical experiments highlighted the importance of MAPK pathway in this setting (116). Selumetinib (i.e., MEK inhibitor) also showed interesting *in vitro* results when associated with sotorasib as assessed by complete inhibition of ERK phosphorylation in H23 *MET*-amplified sotorasib-resistant cells (108) (**Figure 3**). Adachi et al. reported *in vitro* efficacy when targeting IGF1R and FGFR1, RTKs that mediate acquired resistance to *KRAS G12C* inhibitors through EMT (109). Combined treatment with sotorasib, linsitinib (IGF1R inhibitor) and infigratinib (FGFR1 inhibitor) markedly decreased phosphorylation of AKT, ERK and S6 in H358-sotorasib resistant cells (109). Likewise, cell proliferation was significantly decreased with this triple-combination treatment compared to bitherapies of sotorasib with either linsitinib or infigratinib (109) (**Figure 3**).

Overall, these pre-clinical studies highlight that targeting the specific RTK that are involved in by-pass signaling pathways might be a promising strategy to overcome acquired resistance to *KRAS G12C* inhibitors. However, the clinical efficacy of such therapeutic strategies seems difficult to prove in specifically dedicated clinical trials, due to the small proportion of patients who present these alterations. Moreover, mechanisms of acquired resistance to *KRAS G12C* inhibitors seem highly heterogeneous and might co-occur, thereby providing rationale for the instauration of combination treatments at baseline rather than at disease progression.

### Targeting SHP2 and SOS1: Novel Emerging Strategies to Overcome Acquired Resistance to KRAS G12C Inhibitors

SHP2 (Src Homology Phosphatase 2), encoded by *PTPN11*, is a protein tyrosine phosphatase that interacts with activated RTKs either directly by binding with phosphorylated tyrosine residues or indirectly through scaffolding proteins such as GAB1-3, FRS-2 and IRS1-4 (117–119). SHP2 promotes RAS activation pathway, in particular through SOS1 activation (120), and interacts with SRC kinase (121), thereby promoting its activation and subsequent RAS signaling pathway activation (**Figure 3**).

Pre-clinical *in vivo* studies highlight that *KRAS-*mutant NSCLC depends on SHP2 during carcinogenesis (93, 122, 123). Based on these observations, it has been reported that combined therapy with either sotorasib (83) or adagrasib (93) and RMC-4550 enhanced efficacy compared to single monotherapy. Indeed, RMC-4550 combined with AMG-510 achieved high synergy score in both H358-monolayer cell line and CT-26 *KRAS G12C* spheroid models (i.e., synergy score 22.8 and 11.7 respectively) (83). RMC-4550 combined with MRTX849 decreased ERK phosphorylation *in vitro* and in xenograft models of oesophageal cancer (KYSE-410) and NSCLC (H358) (93). Likewise, combined therapy of RMC-4550 plus MRTX849 resulted in significantly greater anti-tumor efficacy in 4 out of 6 *KRAS G12C in vivo* models, compared to monotherapy (93). Notably, results of phase 1 first-in-human clinical trial showed that RMC-4630 led to a disease control rate of 71% in *KRAS G12C* mutated NSCLC patients (n=19), as a single-agent monotherapy (124). Likewise, TNO-155 is currently being tested in a phase 1 dose escalation/expansion trial (NCT03114319) in adults with advanced solid tumors alone (125) or in combination with JDQ443 (KRAS G12C inhibitor), in *KRAS G12C* patients with advanced solid tumors (NCT04699188).

In the setting of acquired resistance to *KRAS G12C* inhibitors, SHP099 (109) and TNO-155 (108) have demonstrated interesting results to overcome acquired resistance to sotorasib (**Figure 3**). SHP099 was found to significantly reduce tumor growth in patient-derived orthotopic xenografts and *KRAS* mutant NSCLC patient-derived xenograft models (122). In EMT-driven, sotorasib-resistant H358 cells, characterized by FGFR1 and IGF1R-ISR1 upregulation, combined therapy with sotorasib, GDC-0941 (PI3K inhibitor) and SHP099 efficiently suppressed PI3K-AKT, MAPK and S6 phosphorylation (109). Similar results were observed in sotorasib-insensitive SW1573 and LU99 *KRAS-*mutated, mesenchymal-like cells (109). Consistently with these observations, combined treatment with sotorasib, GDC-0941 and SHP099 was found to reduce tumor growth and inhibited AKT and ERK signaling pathways in sotorasib-resistant xenograft models (109). Notably, only the combination of these three treatments reversed acquired resistance to sotorasib *in vivo*, compared to bitherapies with sotorasib and either GDC-0941 or SHP099 (109) (**Figure 3**). These results further support that targeting either downstream or upstream effectors of *KRAS G12C* might not be efficient enough to overcome resistance. TNO-155 is another allosteric SHP2 inhibitor (126) that demonstrated synergistic efficacy with an early-stage compound (82) of adagrasib (i.e., compound 12) in H2122 and H1373 *KRAS* mutant NSCLC cells (127). Combined therapy with compound 12 and TNO-155 inhibited ERK phosphorylation and impaired cell proliferation in these models (127). Interestingly, combined treatment of sotorasib and TNO-155 in *MET*-amplified, H23-sotorasib resistant cells inhibited ERK phosphorylation and decreased the rate of RAS-GTP levels, compared to single agent treatment (108) (**Figure 3**).

SOS1 is a key guanine nucleotide exchange factor for *KRAS* activation. SOS1 can either bind to *KRAS* in its GDP-bound state at its catalytic site or binds to *KRAS* in its GTP-bound state at its allosteric binding site, thereby promoting *KRAS* activation (128) (**Figure 3**). SOS1 is also associated in *RAS*-mutated carcinogenesis (129). SOS1 is a promising target in *KRAS-*mutated cancers as it plays a central role in feedback MAPK reactivation (130, 131). Notably, activated MAPK kinases were found to selectively phosphorylate the C-terminal tail of SOS1, but not its paralog SOS2, thereby uncoupling Grb2-SOS1 complex with membrane-bound receptor and activating *RAS* pathway (130, 131). Based on high-throughput screening and structure-based optimization, BI-3406 was recently developed as a potent and selective SOS1 inhibitor (132). BI-3406 bounds specifically to the catalytic site of SOS1, thus blocking SOS1 interaction with GDP-bound *KRAS* (132). Interestingly, pre-clinical studies on BI-3406 reveal that therapeutic efficacy was not limited to *KRAS G12C*-mutant models, as it also induced tumor growth inhibition in xenograft models of *KRAS G12C* (MIA PaCa-2 cells), *KRAS G12V* (SW620 cells), *KRAS G13D* (LoVo cells) and *KRAS G12S* (A549 cells) (132). Moreover, BI-3406 was found to prevent adaptative resistance to MEK inhibition in several *KRAS-*mutated cancer cell lines, and prevented ERK activation rebound following sotorasib treatment *in vitro* (132).

Interestingly, BI-3406 demonstrated promising pre-clinical results to overcome acquired resistance to *KRAS G12C* inhibitors, when combined with treatments that target downstream *KRAS G12C* effectors. BI-3406 or TNO-155 monotherapy did not restore sensitivity to *KRAS G12C* inhibitors in Ba/F3 cells harboring *KRAS G12C* and *Y96S* or *Y96D* secondary mutations (103). In contrast, H358 cells (*KRAS G12C*), modified to express *KRAS Y96S* or *KRAS Y96D* resistant mutations, were sensitive to the combination of BI-3406 and trametinib in both monolayer and 3D cell line models (103) (**Figure 3**). Combined treatment with sotorasib and BI-3406 markedly decreased RAS-GTP levels and inhibited ERK phosphorylation, while AKT activation was less altered, in *MET*-amplified sotorasib-resistant H23 cells (108) (**Figure 3**).

Overall, these studies evidence promising pre-clinical rationales to revert or delay the emergence of acquired resistance to sotorasib and adagrasib (**Figure 3**). Several clinical trials are currently ongoing to evaluate the efficacy of *KRAS G12C* inhibitors in combination with chemotherapy (133, 134) or with targeted therapies including cetuximab, afatinib, pembrolizumab and SHP2, mTOR, CDK4/6 inhibitors (91, 135, 136). These results would be of interest to evaluate whether combined treatments increased response rate to *KRAS G12C* inhibitors, thereby contributing to prevent or delay acquired resistance. Of note, clinical trial evaluating combined treatment with mTOR or CDK4/6 inhibitors (135) will be interesting as these alterations have been recently evidenced to promote acquired resistance to sotorasib in clinical setting (90). As PI3K-AKT pathway is less dependent on RAS pathway and less sensitive to *KRAS G12C* inhibitors, combined strategies targeting upstream effectors (i.e., SOS1 or SHP2) and PI3K-AKT-mTOR pathway seem particularly relevant.

## Immunotherapy in *KRAS*-Mutant NSCLC

Based on tobacco history, high TMB, high levels of PD-L1 expression and pro-inflammatory microenvironment of *KRAS*-mutant NSCLC patients, *KRAS* mutated NSCLC patients are expected to benefit from immune checkpoint inhibitors (ICIs) (137, 138), and this has been demonstrated in *KRAS G12C*-mutant patients in particular (139–141). In IMMUNOTARGET study, among 271 KRAS mutated patients, where immunotherapy was administered as monotherapy in advanced line, response rate was 26% with higher proportion of long responders (12-months PFS: 25.6%) as compared with other oncogenic-driven NSCLC (142). No significant difference has been observed between *KRAS* mutation subtypes in term of response or PFS (142) although *KRAS G12C* patients tend to have higher response rate to ICIs (26.9%) and a longer mPFS (4.0 months) compared with non-*KRAS G12C* (response rate: 18.8% and mPFS 2.9 months) (141). However, as expected, PFS positively correlated with PD-L1 expression. In the Flatiron database of PD-L1≥50% NSCLC, among patients receiving first-line ICI monotherapy, *KRAS* mutations (versus *KRAS* wt) were associated with significant superior survival (mOS,21.1 vs 13.6 months) (137).

In this context, co-occurring mutations might counterbalance clinical benefit of ICIs. While *TP53* co-mutation seems to be associated with a better response to ICIs, *STK11* and *KEAP1* co-mutations seem to impair response to ICIs. Indeed, *TP53/KRAS* co-mutated patients present higher PD-L1 expression, increased CD8^+^ TILs (143) and significantly higher mutational load, compared with either *STK11* or *KEAP1* co-mutated patients. Moreover, patients harboring *TP53/KRAS* mutations were reported to have prolonged PFS to anti- PD-1 therapy compared to *TP53* and *KRAS* wild-type patients (143). In contrast, *STK11* and *KEAP1* co-occurring mutations are associated with resistance to ICIs in *KRAS*-mutant patients, independently of PD-L1 expression (37, 144, 145). Indeed, *post-hoc* analysis of the IMPOWER 150 Phase III trial showed that PFS and OS were markedly decreased for patients harboring *KRAS*, *STK11* and/or *KEAP1* co-mutations treated with carboplatin-paclitaxel combined with either atezolizumab, bevacizumab or atezolizumab and bevacizumab regimen compared to patients with *STK11* and *KEAP1* wild-type (145). *KRAS/STK11* co-mutation might drive intrinsic resistance to PD-1/PD-L1 inhibitors, as it was the only marker associated with PD-L1 negativity among 924 intermediate/high TMB lung adenocarcinoma patients (144). Likewise, *KRAS/STK11* co-mutated tumors are usually considered as “cold-tumors” with paucity of CD3*^+^*, CD4*^+^* and CD8*^+^* TILs (146). *KEAP1* loss downregulates the STING pathway through its interaction with NRF2 (i.e., Nuclear Factor Erythroid 2-like 2) (146). Indeed, *KEAP1* loss mediates the degradation of NRF2, a transcriptional factor that is highly involved in cellular antioxidant pathway (147). It was recently reported that patients with high PD-L1 expression (i.e., ≥50%) had significantly higher levels of stromal-SHP2 compared to those with PD-L1<50% (*p*=0.039) (148). In particular, a significantly higher expression of CD8^+^/CD4^+^ T-cells and CD64^+^ macrophages was observed in stromal compartment of patients with high stromal SHP2 expression compared to those with low stromal SHP2 expression (148). Consistently with these observations, despite small sub-group size, sub-groups analysis showed that patients with high SHP2 expression and PD-L1≥1% had significantly prolonged PFS and OS (148).

In the context of *KRAS G12C* inhibitors, pre-clinical studies highlighted that either sotorasib or adagrasib have enhanced anti-tumor efficacy when combined with anti- PD-1/PD-L1 therapy. In contrast, although either AMG-510 or anti- PD-1 monotherapy caused tumor complete regression in only one out of ten mice, for each therapy; combined treatment achieved a complete and prolonged response in 9/10 CT-26 *KRAS G12C* mice (83). Notably, treatment response was maintained 112 days following treatment arrest. Subsequently, mice that were cured by AMG-510 and anti- PD-1 were then rechallenged with tumor inoculum and showed no tumor reformation (83). Consistently with these observations, co-treatment with adagrasib and anti- PD-1 demonstrated complete and prolonged response *in vivo*, without tumor regrowth after tumor cell inoculation, and increased PFS in *KRAS G12C* genetically engineered mouse models, compared with single-agent monotherapy (94). Based on these pre-clinical results, several clinical trials are currently ongoing evaluating anti- PD-1/PD-L1 with either sotorasib (NCT04185883, NCT03600883) or adagrasib (NCT03785249, NCT04613596). Besides, the potential impact of targeting immune pathways, several other combinations are on study targeting upstream (EGFR mAb and TKIs, pan-ERRB inhibitors, SHP2 and SOS1 inhibitors) and downstream signaling pathways (PIK3, ERK/RAF, MEK, mTOR and CDK4/6 inhibitors) (**Table 3**).

**Table 3:** Ongoing clinical trials to target mutant KRAS.

Combination Target	Combination Options	NCT Number
PD1/PD-L1	sotorasib + AMG-404	NCT04185883
	GDC-6036 + atezolizumab	NCT04449874
	sotorasib + atezolizumab	NCT04185883
	adagrasib + pembrolizumab	NCT03785249/NCT04613596
	sotorasib + pembrolizumab	NCT04185883
	LY3537982 + sintilimab	NCT04956640
	JDQ443 + spartalizumab +/-TNO155	NCT04699188
	sotorasib + anti PD1/PD-L1	NCT03600883
EGFR and pan ERBB inhibitors	adagrasib + afatinib	NCT03785249
	sotorasib + afatinib	NCT04185883
	LY3537982 + cetuximab	NCT04956640
	GDC-6036 + cetuximab	NCT04449874
	GDC-6036 + erlotinib	NCT04449874
	LY3537982 + erlotinib	NCT04956640
SHP2 inhibitors	GDC-6036 + GDC-1971	NCT04185883
	sotorasib + RMC4630	NCT04185883/NCT05054725
	adagrasib + TNO155	NCT04330664
	sotorasib + TNO155	NCT04449874
	JDQ443 + TNO155 +/-spartalizumab	NCT04699188
SOS1 inhibitors	adagrasib + BI1701963	NCT04975256
ERK inhibitors	LY3537982 + temuterkib	NCT04956640
Dual ERK/RAF	sotorasib + VS-6766	NCT05074810
Pan-RAS	BI1823911 + BI1701963	NCT04973163
MEK inhibitor	sotorasib + trametinib	NCT04185883
mTOR inhibitor	sotorasib + everolimus	NCT04185883
Aurora A kinase inhibitors	LY3537982 + LY3295668	NCT04956640
CDK4/6 inhibitors	LY3537982 + abemaciclib	NCT04956640
	sotorasib + palbociclib	NCT04185883
Chemotherapy	sotorasib + carboplatin pemetrexed/docetaxel	NCT04185883
Anti-angiogenic	GDC-6036 + bevacizumab	NCT04449874

## Conclusion

Similar other targeted therapies, the biological mechanisms of acquired resistance to *KRAS G12C* are highly heterogeneous. The development of combine therapeutic approaches, based on SHP2 and SOS1 inhibitors, might be a valuable strategy to target both intrinsic and acquired resistance. Moreover, pre-clinical evidence highlights that combined treatment involving *KRAS G12C* inhibitors as well as upstream and downstream effectors is usually necessary to achieve therapeutic efficacy. Although promising, these observations rise concerns on the safety profile of such combined treatments in clinical setting. After long decades of considering *KRAS* as an elusive target in NSCLC, sotorasib and adagrasib as well as emerging *KRAS*-mutant targeted treatments constitute an unprecedent improvement to efficiently target *KRAS*-mutant NSCLC. Besides providing new therapeutic strategies to target *KRAS*-mutant cancer, sotorasib and adagrasib also provide a potential therapeutic rational to overcome *KRAS* secondary mutations mediating resistance to other therapies in other oncogenic driven NSCLC.

## Author Contributions

A-LD, AS, SO-C: contributed to this paper with the design. A-LD, CL, AS, SO-C: literature search. A-LD, CL, AS, SO-C: revision, editing and final approval. All authors contributed to the article and approved the submitted version.

## Funding

A-LD was supported by a 2020–2021 clinical fellowship award for a Master degree from Saint-Etienne Jean Monnet University. CL received a PhD fellowship from La Ligue contre le Cancer (2017–2020). This work was supported by the Institut National du Cancer (INCa) (PRT-K program no. 2018-024 EMT-CoNCEPT).

## Conflict of Interest

AS received honoraria from Amgen for participation in Board meetings.

The authors declare that the research was conducted in the absence of any commercial or financial relationships that could be construed as a potential conflict of interest.

## Publisher’s Note

All claims expressed in this article are solely those of the authors and do not necessarily represent those of their affiliated organizations, or those of the publisher, the editors and the reviewers. Any product that may be evaluated in this article, or claim that may be made by its manufacturer, is not guaranteed or endorsed by the publisher.
